# Pulsed Vacuum Drying of Pepper (*Capsicum annuum* L.): Effect of High-Humidity Hot Air Impingement Blanching Pretreatment on Drying Kinetics and Quality Attributes

**DOI:** 10.3390/foods11030318

**Published:** 2022-01-24

**Authors:** Zhihua Geng, Xiao Huang, Jun Wang, Hongwei Xiao, Xuhai Yang, Lichun Zhu, Xiaochen Qi, Qian Zhang, Bin Hu

**Affiliations:** 1 Engineering Research Center for Production Mechanization of Oasis Special Economic Crop, College of Mechanical and Electrical Engineering, Ministry of Education, Shihezi University, Shihezi 832000, China; gzhjdxy@163.com (Z.G.); dushuxiaoyang@163.com (X.H.); jun.wang@nwafu.edu.cn (J.W.); xhwcaugxy@163.com (H.X.); zhulichun0204@126.com (L.Z.); qxcaxt2@163.com (X.Q.); zqq80@163.com (Q.Z.); 2 College of Food Science and Engineering, Northwest A&F University, Xianyang 712100, China; 3 College of Engineering, China Agricultural University, Beijing 100083, China; 4 Xinjiang Production and Construction Corps Key Laboratory of Modern Agricultural Machinery, Shihezi 832000, China

**Keywords:** high-humidity hot-air impingement blanching (HHAIB), pulsed vacuum drying (PVD), drying characteristics, kinetic modeling, transmission electron microscopy (TEM), polyphenol oxidase activity

## Abstract

With a high moisture content, fresh peppers are perishable and rot easily. Drying is essential for shelf-life extension. The natural thin wax layer on the pepper surface hinders moisture transfer. Traditionally, chemical dipping or mechanical pricking is used to remove this wax layer. However, in chemical dipping, chemical residues can trigger food-safety issues, while the low efficiency of mechanical pricking hinders its industrial application. Feasible pretreatment methods are advantageous for industrial use. Here, an emerging pretreatment technique (high-humidity hot-air impingement blanching, HHAIB) was used for peppers before drying and its effects on drying characteristics, microstructure, and polyphenol oxidase (PPO) activity were explored. The impact of drying temperature on color parameters and red pigment content of pulsed-vacuum-dried peppers was also evaluated. PPO activity was reduced to less than 20% after blanching at 110 °C for 60 s. HHAIB reduced drying time and PPO activity and promoted chemical-substance release. Effective water diffusivity was highest (5.01 × 10^−10^ m^2^/s) after blanching at 110 °C for 90 s, and the brightness value and red pigment content were highest (9.94 g/kg) at 70 °C. HHAIB and pulsed vacuum drying are promising pretreatment and drying methods for enhancing the drying rate and quality of red peppers.

## 1. Introduction

Peppers (*Capsicum annuum* L.) are annuals or limited perennials belonging to the nightshade family [1] and are popular for their bright color and pungent taste. According to Food and Agriculture Organization (FAO) statistics, the global pepper production is approximately 3.83 × 10^7^ tons. With production of 1.90 × 10^7^ tons, China ranks first worldwide, followed by Mexico (3.24 × 10^6^ tons), the European Union (2.81 × 10^6^ tons), and Turkey (2.63 × 10^6^ tons) [2]. Freshly picked peppers show high respiratory and enzymatic activity [3], with high moisture contents of up to 60–85%, which greatly increases their susceptibility to microbial infection [4]. Fresh peppers spoil and rot within 2–3 days if not processed in time, leading to a 12–15% loss in production [5]. Therefore, it is critical for the pepper industry to extend the shelf life of peppers and reduce post-harvest losses.

Drying is the most widely used and economically viable method to improve the shelf life of peppers [6]. Peppers naturally form a waxy layer on their skin during growth, which provides protection from external microbial infection and UV damage [3]. However, this layer prevents moisture diffusion during the drying process, thus leading to difficulties in drying: prolonged drying time; increased energy consumption and biochemical reactions, oxidative degradation, and physical changes in the function of pepper enzymes; and serious quality deterioration [5]. Therefore, pretreatment to eliminate the enzymes and improve product quality has become an indispensable step in pepper processing [7].

The traditional pretreatment methods used before drying peppers include chemical dipping pretreatment [8]; hot-water [9], steam, ohmic, and infrared and microwave [10] blanching; and mechanical pricking. Chemical-solvent soaking can increase the pepper-drying speed, shorten the drying time, and improve product quality. However, this method can lead to chemical residues that cause food-safety problems, contradicting the current trend of green consumer protection [8]. The equipment for hot-water blanching requires low investment and is easy to operate. However, this method involves processing at high temperatures for a long period and may result in considerable loss of heat-sensitive material. Moreover, waste-liquid treatment after blanching is a difficult and costly process. Steam blanching can effectively solve the problem of nutrient loss and waste-liquid pollution caused by traditional hot-water blanching. However, the heat transfer coefficient of traditional steam blanching is not high and the blanching efficiency is low [11]. The ohmic, infrared, and microwave blanching methods offer advantages, such as a short heating period, high drying rate, and high efficiency. However, these blanching methods generate oxygen, which, in turn, promotes nutrient oxidation. Moreover, the water content in peppers may evaporate, and high-intensity physical treatment can cause cell folding and damage the product’s microstructure [12].

In the case of mechanical pricking, Arora et al. [13] observed that the drying time of peppers after perforation was shortened by 12–48% compared with that without perforation. Yong et al. [14] observed that perforation pretreatment could increase the drying rate of peppers and the drying time was closely related to the pore size. However, because of perforation, the structure of the pepper itself is destroyed, leading to the loss of some nutrients, while punching the pepper destroys its original structure and affects the consumer’s desire for consumption. Therefore, it may not be suitable for large-scale industrial application.

High-humidity hot-air impingement blanching (HHAIB) is an emerging and efficient pretreatment method for fruit and vegetables that inactivates enzymes using a combination of air impingement and hot-steam blanching [15]. Compared with traditional hot-water blanching, HHAIB can greatly reduce loss of water-soluble nutrients [16] and has an extremely high convective heat transfer coefficient. HHAIB has been used to blanch broccoli florets, while maintaining their color and avoiding browning [17], and to pretreat apricots to increase their drying rate [16].

Pretreatment can increase the drying rate to ensure drying quality but needs to be combined with advanced drying methods, especially for the retention of red pigments and other vibrant colors in the peppers. At present, open-sun drying and hot-air drying are commonly used for drying peppers. Open-sun drying has problems, such as susceptibility to weather, condensation, and dew at night, which is conducive to microorganisms, increases susceptibility to dust and insect feces contamination [18], prolongs drying time, and creates a need for large drying sites [19]. Studies have shown that peppers lose up to 80% of carotenoids during natural drying [20]. Hot-air drying, although low cost and applicable to shortening the drying time, involves prolonged exposure to a high-temperature environment and causes oxidative degradation of heat-sensitive components, thus reducing the quality and active ingredient extraction rate of chili peppers [19]. The higher the temperature, the lower the rehydration ratio, vitamin C content, and total phenolic content; Scala et al. [21]. showed that hot-air drying resulted in an 82% loss of carotenoids and an 88% loss of vitamin C in peppers. Pulsed vacuum drying (PVD) uses constantly changing vacuum pressure in the drying chamber to enhance moisture transfer and accelerate the drying process [22]. PVD involves alternate cycling of vacuum and atmospheric pressure, with little oxygen present in the drying chamber. Thus, PVD can reduce browning reactions, oxidation deterioration, and other adverse biochemical reactions, thus improving the quality of dried products [23,24]. The pressure cycle promotes the formation of porous structures in the peel and interconnects and enlarges the micropores in the dried products [23]. Simultaneously, the pressure cycle can generate fissured structures, promoting water circulation and accelerating drying [25]. Thus, PVD has several advantages and, in recent years, has been widely used to dry fruit and vegetables, including green prickly ash [26], blueberries [23], lemons [27], grapes [28], wolfberries [29], pollen [30], ginger [31], poria [32], and berries [33]. However, to the best of our knowledge, no studies have detailed the effects of HHAIB on the drying characteristics and PVD of peppers.

Drying is a complex process involving heat and mass transfer [34] and is influenced by material characteristics, media parameters, process parameters, and environmental conditions [35]. With the development of drying technology and scientific progress, using mathematical models to describe and predict the drying process has become an important part of drying research [36]. Thus, establishing a mathematical model of the drying process can facilitate analysis and evaluation of the entire drying process, optimize the drying parameters, predict the drying end point, and improve the quality of the dried product [37].

The objectives of the current study were to (1) explore the effect of HHAIB on PVD kinetics and quality attributes, including red pigment content and color parameters of peppers; (2) investigate ultrastructural changes in peppers under different blanching conditions and elucidate the mechanisms underlying the changes in macroscopic properties; and (3) identify a suitable kinetic model for drying peppers, which is necessary for selecting suitable pretreatment and drying technology for red pepper drying.

## 2. Materials and Methods

**Figure 1:**
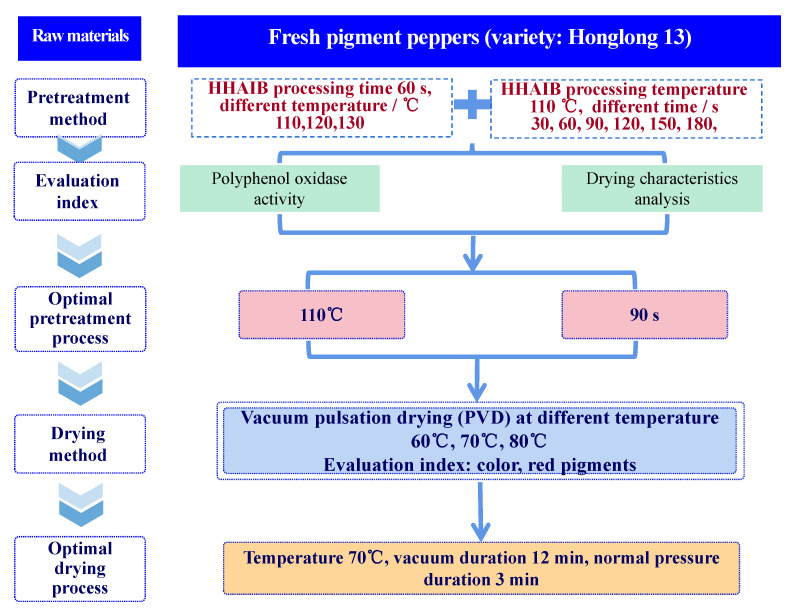
Schematic overview of the experimental setup.

The overall experimental setup process is shown in Figure 1.

### 2.1. Materials

Fresh peppers (variety: Honglong 13) without mechanical or insect damage were picked on 27 August 2019, from No. 124 State Farm in Gaoquan (Kuitun, Xinjiang, China). To ensure uniformity in the physical characteristics of the materials, we selected fresh red peppers of the same size with undamaged surfaces (average length, 13 ± 1.5 cm; average weight, 17 ± 0.9 g) for the blanching and drying experiments. The initial moisture content of the peppers was 84.98 ± 0.8% (wet basis), as measured using a vacuum drying method (drying for 24 h at 70 °C; vacuum degree, 6 kPa). All peppers were refrigerated (temperature, 4 ± 1 °C; relative humidity, 90 ± 5%) 1 d before the experiment. The fresh peppers were removed from the refrigerator, cleaned using tap water, drained, dried using absorbent paper, and kept indoors at room temperature (20 °C) for 30 min. Subsequently, the stems and seeds were removed, the peppers were cut into 4 × 1.2 cm slices, and the slices were laid flat on stainless steel mesh trays (weight per tray, 100 ± 3 g).

### 2.2. HHAIB Pretreatment Experiments

**Figure 2:**
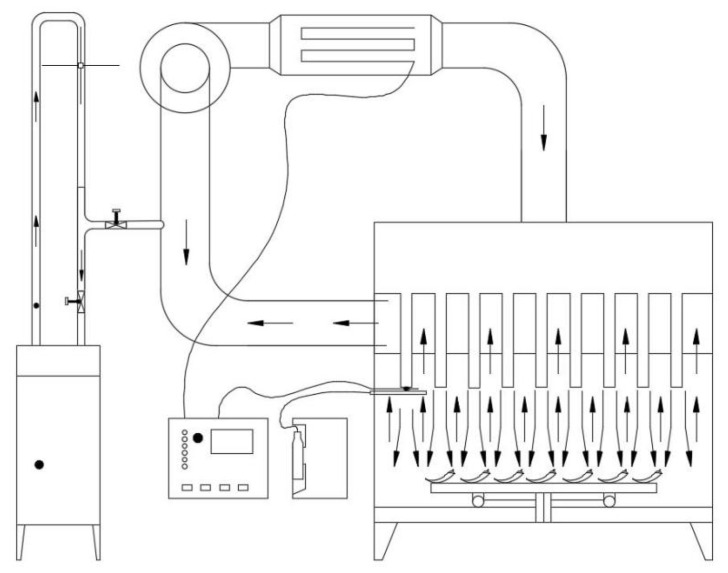
Schematic diagram of equipment used for high-humidity air impingement blanching.

The characteristics of the HHAIB device (Figure 2; designed by the College of Engineering of China Agricultural University) were reported in detail in our previous work [11]. The device is composed of electric heating pipes, steam generators, nozzles, centrifugal fans, and controllers, and other components [16]. It generates steam from a steam generator; a centrifugal fan provides circulating air flow, sprays it to the surface of the material through a circular nozzle, and controls the blanching temperature through a proportional-integral-derivative (PID) controller (Omron, model E5CN, Tokyo, Japan) [38]. Based on pre-experiments, an air velocity of 14.0 ± 0.5 m/s and a blanching relative humidity of 35–40% were the optimal conditions for pepper pretreatment. Fresh peppers were used as the control group, and peppers in the other groups were exposed to blanching temperatures of 110, 120, or 130 °C for blanching durations of 30, 60, 90, 120, or 150 s. Each treatment was performed in triplicate.

### 2.3. Drying Process

#### 2.3.1. PVD Equipment

**Figure 3:**
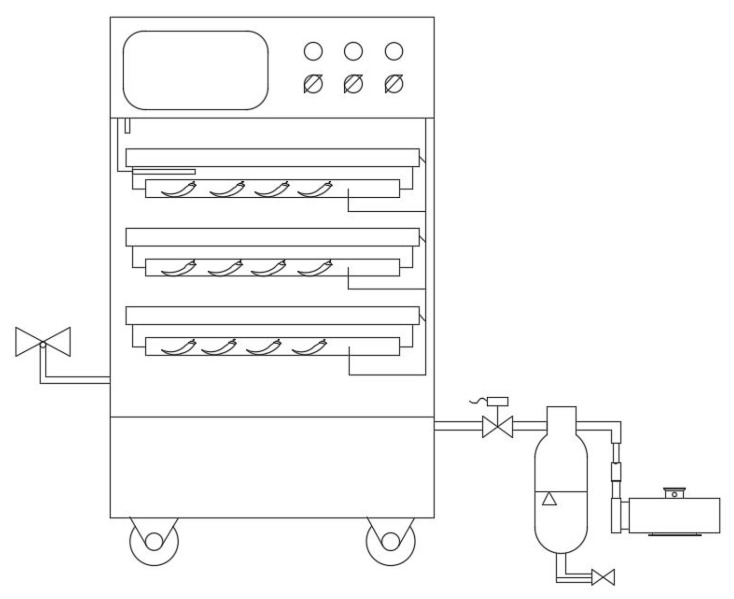
Schematic diagram of the device used for drying.

We used PVD to dry both treated and untreated pepper samples. The PVD equipment (Figure 3) was also obtained from the College of Engineering of China Agricultural University. For PVD, the distance between the upper and lower heating plates was 5 cm, the vacuum degree was 8.0 kPa, the holding time of atmospheric pressure was 3 min, the holding time of the vacuum was 12 min, and the vacuum and atmospheric pressures were cycled alternately. The working time of the dryer (from the atmospheric pressure to the vacuum state) was approximately 40 s. The temperature and humidity in the dryer were maintained at constant levels.

#### 2.3.2. Experimental Procedure

We set the temperature and humidity of the superheated steam before the pretreatment. As soon as the heating plate reached and maintained the set value, the peppers were placed into the blanching room for the blanching test. The distance from the HHAIB nozzle to the pepper surface was 9 ± 0.1 cm, the wind speed was 14.0 ± 0.5 m/s, and the indoor relative humidity was 35–40%. The peppers were laid flat on the material tray in one layer to avoid overlapping. After blanching, the peppers were tested using the PVD method (drying temperature, 70 °C; vacuum time, 12 min; normal pressure time, 3 min). During the drying process, weight loss was measured every 30 min using an electronic balance (SP402, Ohaus Co., Parsippany, NJ, USA). The drying test was stopped when the final dry basis moisture content was lower than 0.11 g/g [19,39]. The dried peppers were cooled to room temperature (20 °C), packed using a vacuum packing machine, and stored at room temperature and away from light for further tests.

### 2.4. Measurement of Polyphenol Oxidase (PPO) Activity

PPO activity was measured as reported previously [10]. The enzyme extracting solution was prepared as follows: 5 g of fresh pepper sample was added to 5.0 mL of an extraction buffer (including 1 M MPEG, 4% PVPP, and 1% Triton X-100), homogenized in an ice bath, and centrifuged at 4 °C and 12,000× *g* for 30 min. The supernatant was collected and used as the enzyme extracting solution. We rapidly mixed 4.0 mL of acetic acid–sodium acetate buffer (50 mmol/L; pH, 5.5), 1.0 mL of pyrocatechol (50 mmol/L), and 0.03 mL of the enzyme extracting solution in a 10 mL centrifuge tube and loaded this in a cuvette within 15 s. The cuvette was placed in a spectrophotometer (Beijing Purkinje General Instrument Co. Ltd., Beijing, China) sample room for measurement. Distilled water was used as the reference, and absorbance at 420 nm wavelength was recorded as the initial value. Data were recorded every 1 min at no less than 6 points, and the measurement was repeated three times. We then used 3–4 datapoints with the best linearity to calculate the slope and measure enzymatic activity. Each increment of 1 in the absorbance change in 1 min (using 1 g of fresh pepper sample) equaled 1 peroxidase unit, ΔOD_420_/min·g, as shown in Equation (1):

(1) $$U=\frac{\Delta OD_{420}\times V}{V_{s}\times m}$$

where *U* is PPO activity, Δ*OD*_420_ is the slope with the best linearity in absorbance changes per min, *V* is the total volume of the sample extract (mL), *V_s_* is the volume of the sample extract during testing (mL), and *m* is the mass of the sample (g).

### 2.5. Analysis of Drying Characteristics

#### 2.5.1. Moisture Ratio (MR)

The *MR* of peppers was calculated according to Equation (2), as reported previously [19,40]:

(2) $$MR=\frac{M_{t}\;-\;M_{e}}{M_{0}\;-\;M_{e}}$$

where *M_t_* is the dry basis moisture content of red peppers (kg/kg) at time *t*, *M*_0_ at *t *= 0 is the initial dry basis moisture content of red peppers (kg/kg), and *M_e_* is the equilibrium moisture content (kg/kg).

#### 2.5.2. Drying Rate (DR)

The *DR* of peppers was calculated according to Equation (3), as reported previously [41]:

(3) $$DR=\frac{M_{t_{1}}-M_{t_{2}}}{t_{2}-t_{1}}$$

where *M_t_*_1_ is the dry basis moisture content (g/g) at time *t*_1_ and *M_t_*_2_ is the dry basis moisture content (g/g) at time *t*_2_.

#### 2.5.3. Effective Moisture Diffusivity (*D*_eff_)

*D*_eff_ was measured according to Equation (4), as reported previously [19,42]:

(4) $$D_{\mathrm{eff}}=\frac{D_{\mathrm{cal}}}{R_{g}}$$

where *D*_cal_ is the estimated effective moisture diffusivity (m^2^/s) and *R*_g_ is the physical dimension constant.

### 2.6. Kinetic Modeling

Mathematical models are a good representation of research problems and different laws and inter-relationships between parameters and can be used to analyze the relationship between parameters and to predict trends. Several common thin-layer drying models were selected to fit the drying process in this study.

Modified Weibull: The Weibull model is widely used for describing moisture changes in food materials under different drying conditions. The modified Weibull distribution function was calculated using Equation (5) [43]:

MR = exp(−(*t*/*α*)*^β^*) − A, (5)

where *t* is the drying time, *α* is the scale parameter of the Weibull model, and *β* is the shape parameter of the model.

The Lewis [44] distribution function was calculated using Equation (6):

MR = exp(−*k* × *t*), (6)

where *k* is the rate constant and t is the drying time.

The Page [45] distribution function was calculated using Equation (7):

MR = exp(−*k* × *t^n^*), (7)

where *k* and *n* are the rate constants and t is the drying time.

The Wang and Singh distribution function [46] was calculated using Equation (8):

MR = 1 + *a* × *t* + *b* × *t*^2^, (8)

where *a* and *b* are the rate constants and t is the drying time.

The two-term distribution function was calculated using Equation (9) [47]:

MR = *a* × exp(−*k*_0_ × *t*) + *b* × exp(−*k*_1_ × *t*), (9)

where *a*, *b*, *k*_0_, and *k*_1_ are the rate constants and t is the drying time.

The degree of fit between each model and the data was evaluated using the coefficient of determination (*R*^2^), the root mean square error (RMSE), and *χ*^2^, which were calculated using Equations (10)–(12) [48]:

(10) $$R^{2}=1\;-\;\frac{\sum_{i=1}^{N}(MR_{\mathrm{pre},i}-MR_{\exp,i}{)}^{2}}{\sum_{i=1}^{N}({\bar{MR}}_{\mathrm{pre},i}-MR_{\exp,i}{)}^{2}}$$

(11) $$RMSE={\left[\frac{1}{N}\sum_{i=1}^{N}{\left(MR_{\mathrm{pre},i}-MR_{\exp,i}\right)}^{2}\right]}^{\frac{1}{2}}$$

(12) $$\chi^{2}=\frac{\sum_{i=1}^{N}{\left(MR_{\exp,i}-MR_{\mathrm{pre},i}\right)}^{2}}{N-z}$$

where *MR*_exp,i_ and *MR*_pre,i_ are the experimental and computed dimensionless moisture ratios, respectively; *N* is the number of experiences; and *z* is the number of constants.

### 2.7. Color Measurement

The color parameters of the dried peppers were measured using a LabScan XE spectrophotometer (HunterLab, Reston, VA, USA). The dried peppers were ground into powder and screened using a standard 28-mesh sieve. Using a spectrophotometer, the color of the paprika red pigment was measured in triplicate for each group and the average was calculated. The luminance (L*) and green/red value (a*) were determined according to the CIELAB color system (or L* a* b* color system), and the L* and a* column diagrams of ground paprika were drawn. In the diagram, L* indicates the luminance (black: L* = 0; white: L* = 100) and a* indicates the green/red value (range, −60 (pure green) to +60 (pure red)) [49]. The higher the +a*, the redder the color, whereas the lower the −a*, the greener the color.

### 2.8. Determination of Red Pigment Content

The red pigment content of peppers was measured according to the ISO guidelines [50]. The pepper sample (100–200 g) was powdered and screened using a 28-mesh sieve (sieving rate, >99%). A sample of this (0.1 g, accurate to ±0.0002 g) was added to a 250 mL flask containing 200 mL acetone. The flask was placed on a shaker and incubated for 4 h in the dark. Following this, the flask was tilted slightly to allow the paprika to settle at the bottom of the flask. The solution was diluted with acetone to the calibration standard, shaken, and allowed to rest for 10 min. A graduated pipette was used to transfer the supernatant into a cuvette. Acetone was used as the reference liquid, and absorbance at 460 nm wavelength (*A*_460_) was measured using a spectrophotometer.

The total pigment content of ground paprika (c) was measured as the g weight of red pigment in 1 kg of dry sample and was calculated (ISO 1989) using Equation (13):

(13) $$c=\frac{A_{460}\times f\times 2.5\times 10^{5}}{2250\times (100-H)\times m}$$

where *A*_460_ is the absorption of the test solution, *f* is the calibration factor for the spectrophotometer (0.98 in this study), 2.5 × 10^5^ is the conversion coefficient, 2250 is the absorption coefficient of red pigment in natural paprika, *H* is the sample moisture content (mass fraction, %), and m is the sample mass (g).

### 2.9. Ultrastructure Analysis

The pepper ultrastructure was analyzed using transmission electron microscopy (TEM) as follows [51]. A scalpel was used to obtain pericarp tissue (size, 2 × 2 mm) from the epidermis of the red peppers. The sections were fixed with 5% glutaraldehyde and 4% paraformaldehyde in 0.1 M sodium phosphate buffer (pH 7.2) for 2 h. After three 15-min washes with the buffer, the samples were post-fixed in 1% osmium tetroxide in the same buffer for 2 h. The samples were then immersed in Spurr resin overnight at 4 °C to allow infiltration, and then embedded in Spurr resin. The blocks were sectioned on a Leica EM UC6 ultramicrotome (Leica Microsystems, Wetzlar, Germany). The sections were collected on copper grids and stained with uranyl acetate, followed by lead citrate. The sections were then examined using Hitachi H-7650 TEM (Hitachi High-Tech Corporation, Tokyo, Japan).

### 2.10. Statistical Analysis

The data are expressed as the mean and standard deviation of three replicate measurements. An optimal experimental design was used based on a single factor test [52]. The data were analyzed using analysis of variance and Duncan’s multiple range test with SPSS statistical software (version 21.0. IBM Corp., Armonk, NY, USA). Differences were considered statistically significant at *p* < 0.05.

## 3. Results

### 3.1. Effects of Different Blanching Methods on the Residual Activity of PPO

**Figure 4:**
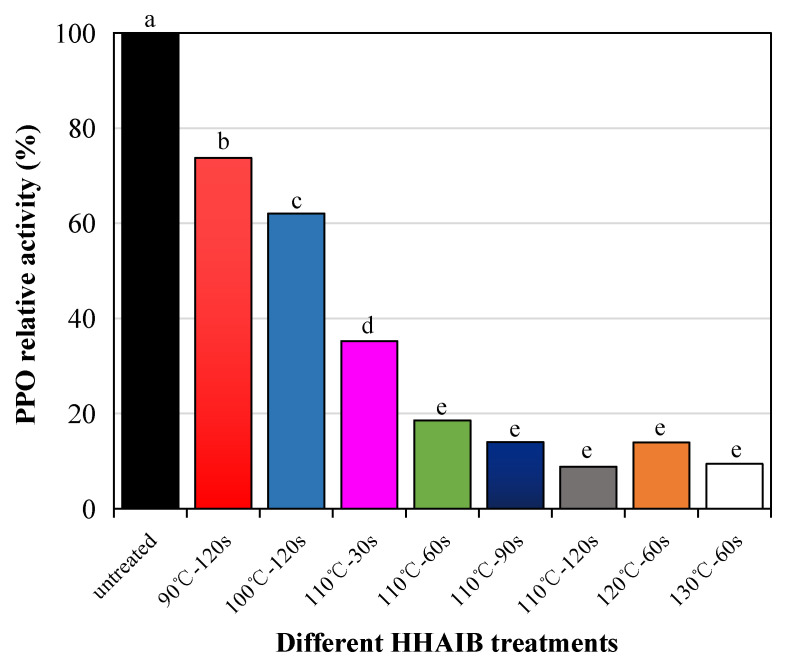
Effects of HHAIB (high-humidity hot-air impingement blanching) on residual activity of PPO (polyphenol oxidase). Notes: different letters in the figure reveal significant differences (*p* < 0.05) according to the Duncan test.

PPO promotes biochemical reactions, and its activity is closely related to changes in product quality; therefore, PPO activity is often used as an indicator of the effects of blanching [53]. In this study, PPO activity was 73.75% after blanching at 90 °C for 120 s and 62.05% after blanching at 100 °C for 120 s. This indicates that blanching temperatures of 90 and 100 °C did not rapidly inactivate PPO, which is inappropriate for the blanching pretreatment of pigment peppers. A blanching temperature of 110 °C for a treatment time >60 s can inactivate PPO [54]. Therefore, blanching temperatures of 110, 120, and 130 °C at blanching durations ≥60 s were selected to study the impact of the blanching temperature on the drying kinetics and drying quality of pigment peppers (Figure 4). Nicolas [55] showed that, when the processing temperature exceeded 40 °C, PPO activity was destroyed. Zhu [56] reported that the PPO of apple slices treated at 60–80 °C was also destroyed, but studies have shown that hot-water and other blanching methods require a long time [15]. Deng [15] also showed that HHAIB inactivates PPO when the core temperature reaches 61.1 °C after treatment for 90 s. Wang’s study of peppers found that HHAIB treatment for 2 min could inactivate PPO enzymes, possibly because of differences in the studied pepper varieties [38]. Wang studied red bell peppers [4], while the peppers used in this study were used to extract the pigment, which is more sensitive to temperature, thus providing a good reference for actual hot-pepper blanching, avoiding the loss of nutrients and pigment degradation caused by excessive blanching.

### 3.2. Effects of PVD on the Drying Characteristics of Red Peppers

**Figure 5:**
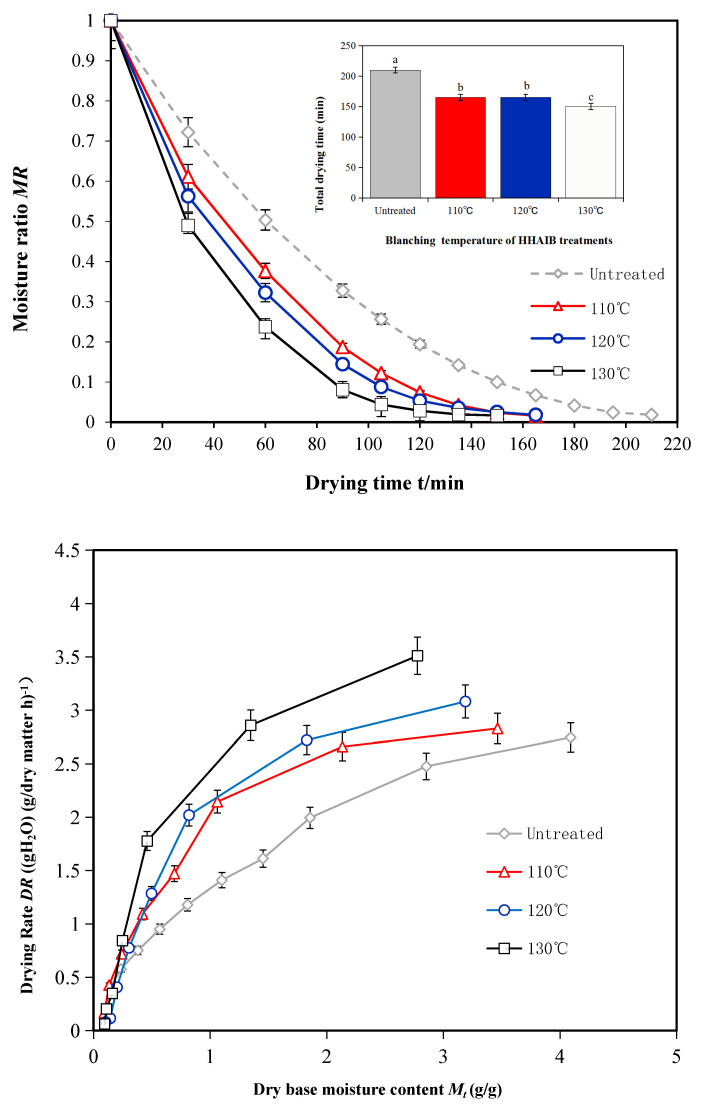
Drying kinetics curve of pepper at different blanching temperatures. Notes: different letters in the figure reveal significant differences (*p* < 0.05) according to the Duncan test.

The changes in MRs with drying time during the PVD test (drying temperature, 70 °C; vacuum duration, 12 min; and atmospheric pressure duration, 3 min) are shown in Figure 5. Peppers not pretreated and those pretreated at different blanching temperatures were tested. The drying time of HHAIB-pretreated peppers was 28.57% shorter than that of peppers without pretreatment, indicating that blanching pretreatment significantly reduced the drying time and energy consumption during drying [5,11,15]. This is because pretreatment with HHAIB removes the waxy layer on the pepper surface and produces tiny gaps on the surface, thus allowing the internal moisture to escape easily [4,5]. In addition, blanching separates the inner cell wall of the pepper and increases the permeability of the cell wall, which allows the flow of internal moisture to the surface and enhances drying efficiency [11,16]. Similar results have been reported by studies investigating the drying pretreatment of seedless grapes and apricots [11,16]. These studies showed that an increase in blanching temperature reduced the drying time to a limited degree. During the latter stages of PVD, the higher the blanching pretreatment temperature, the longer the drying time of pigment peppers [16]. One possible reason for this is that excessively high temperatures destroy the inner tissues of pigment peppers and block the path of escape for moisture, thus preventing the flow of internal moisture to the surface [4,5].

**Figure 6:**
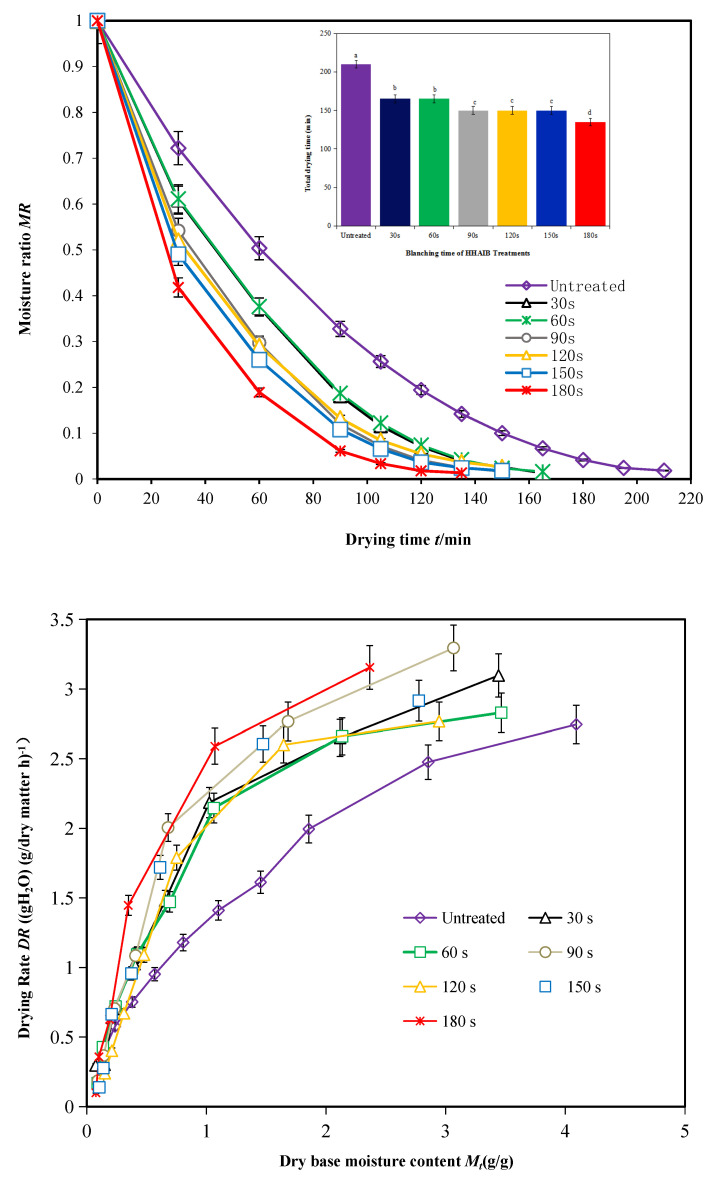
Drying kinetics curve of pigment pepper at different blanching times. Notes: different letters in the figure reveal significant differences (*p* < 0.05) according to the Duncan test.

During PVD, the drying speed decreased with reduction in the dry basis moisture content in peppers (Figure 6) [19]. However, when the initial dry basis moisture content was reduced to 0.5 g/g, the drying speed increased with increasing blanching temperature. The drying speed showed slight differences at different blanching temperatures when the dry basis moisture content was below 0.5 g/g [4]. Overall, the following trends were observed (Figure 6): (a) the drying speed decreased with a decrease in the dry basis moisture content during the PVD of pigment peppers [24]; (b) the drying speed increased with an increase in blanching temperature when the initial dry basis moisture content was reduced to 0.5 g/g; and c) the drying speed was slightly different at different blanching temperatures when the dry basis moisture content was below 0.5 g/g.

Peppers without pretreatment and those pretreated at a blanching temperature of 110 °C for different blanching durations were tested using PVD (drying temperature, 70 °C; vacuum time, 12 min; and atmospheric pressure, 3 min). The moisture diffusion coefficients, as estimated using the modified Weibull distribution function, are listed in Table 1. The moisture diffusion coefficient of pigment peppers without pretreatment was estimated at 4.22 × 10^−10^ m^2^/s. The moisture diffusion coefficient of pretreated pigment peppers first increased and then decreased with increasing blanching duration [4], and reached a maximum of 5.01 × 10^−10^ m^2^/s when the blanching duration was 90 s. This indicates that an increase in blanching duration may accelerate moisture diffusion during PVD [15]. However, a longer blanching duration would also destroy the interior tissues of pigment peppers, block the path of escape for moisture, hinder the flow of moisture to the surface, and further reduce the moisture diffusion coefficient [5]. Wang et al. [4] also found that HHAIB pretreatment could effectively increase the drying speed and reduce the drying time of peppers. However, a longer blanching duration may lead to the collapse of cell structures, bond the interior tissues, and hinder the diffusion of moisture [49], which is consistent with our findings.

### 3.3. Different Models Used to Fit the Drying Parameters

In this study, we applied five drying models to the experimental data of pepper drying (Table 2). We found that the coefficient of determination of the Page model was higher than those of the two-term, modified Weibull, Wang and Singh, and Lewis distribution functions, suggesting that the Page model was a better fit for these parameters. Notably, the size parameter (α) in the modified Weibull model gradually decreased with the increase in drying temperature—a trend also observed for changes in drying time. In a study on the PVD of grapes pretreated with blanching, Bai et al. [49] reported similar results, showing that α, respectively, was 45%, 41%, 56%, 50%, and 57% of the completion time of PVD in the five models. This is because, when using the Weibull model, the MR of the material is set at 1 before the drying process. However, before starting the drying process, blanching pretreatment reduces the dry MR of the material to 80% of the original. Therefore, the α in the modified Weibull model should be 80% of the α in the Weibull model, which is approximately equal to the completion of the drying process in 51% of the time required. The α in our experiment was in line with the results of that study [49]. The shape parameter (β) in the modified Weibull model varied from 0.827 to 0.998, indicating that the material was always in the reduced-speed drying stage during PVD and that the drying temperature had no significant effect on β.

### 3.4. Effect of Drying Temperature on the Pigment Content of Red Peppers

Measuring the changes in color pigment content is a common method to evaluate the quality of drying in pepper [57]. Capsaicin is a natural organic pigment that is nontoxic, provides a strong color, and is stable in nature. Because of these advantages, capsaicin is widely used in the food and cosmetic industries [58]. Capsaicin also has anticancer and antioxidation effects and helps prevent cardiovascular diseases and is, therefore, used in the medicine and healthcare industries [58]. Capsaicin and *C. annuum* have been approved as natural food additives for unlimited use by the FAO, the UK, Japan, the WHO, and other countries and organizations [59,60] and are in great demand in international markets. At present, developing the technology to extract red pigments from paprika and investigating its applications are research areas of interest in many countries [61].

The color characteristics of dried peppers are listed in Table 3. These results indicate that the drying temperature had a significant effect on the color of peppers dried under the same pulsation ratio. With an increase in drying temperature, the L^*^ value of the color parameter of peppers first increased and then decreased. This was due to the degradation of the red pigment and the formation of dark pigments as a result of the Maillard reaction during the drying process, leading to a change in brightness [4]. Thus, the drying temperature had a significant effect on the brightness (L* value) of the color parameter of peppers. The brightness of the pigment peppers was highest (44.43 ± 0.18) at a drying temperature of 70 °C. This indicated that, when the drying temperature was 70 °C, peppers pretreated with blanching showed the least browning and had the best color during PVD. However, a very high drying temperature caused a decrease in brightness. This is consistent with the results of Rhim and Hong [60], who reported that the brightness (L*) of peppers decreases as the drying temperature increases.

After the pepper had been dried, the green–red (a*) and blue–yellow (b*) parameters showed significant changes. An increase in drying temperature promotes the synthesis of capsanthin compounds [19], which can explain the increase in a*. A higher temperature also shortens the drying time and reduces the levels of carotenoids, such as violaxanthin, mutagenic xanthin, and zeaxanthin [22]. Oxidation and the degradation of compounds (such as capsolutin, β-cryptoxanthin, and β-carotene) ensure color retention [60]. Thus, the increase in b* may be due to the low-oxygen environment of the PVD chamber, which inhibited the oxidation of these compounds and helped maintain the yellow color of the dried product [62].

### 3.5. Effect of Drying Temperature on the Red Pigment Content of Red Peppers

Following pretreatment, the red pigment contents of peppers under PVD at different drying temperatures are listed in Table 3. When the vacuum holding time was 12 min, the normal pressure holding time was 3 min, and the drying temperatures were 60 °C, 70 °C, and 80 °C, the red pigment contents after drying were 9.47 g/kg, 9.94 g/kg, and 9.60 g/kg of dried pepper, respectively. Thus, the drying temperature had a significant effect on the red pigment levels. With an increase in drying temperature, the red pigment levels of the peppers first increased and then decreased. This may be because the drying times were shorter for higher drying temperatures, resulting in lower amounts of red pigment loss [19]. However, when the temperature is very high, the red pigment becomes unstable and degrades, and its levels decrease gradually [57]. When the drying temperature was 70 °C, the red pigment content of peppers was the highest and was 4.96% higher than that at 60 °C. This indicates that, at a drying temperature of 70 °C, peppers pretreated with blanching showed the least amount of oxidation and decomposition of red pigment. Studies by Xie et al. [24] and Bai et al. [49] on the PVD of wolfberries and grapes, respectively, also showed that colors deteriorate with an increase in drying temperature [48]. This may be due to strengthening of the Maillard reaction and degradation of carotenoids in high-temperature environments, resulting in a darker color (such as melanoids) [60].

### 3.6. TEM Imaging of Peppers with Different Blanching Treatments

**Figure 7:**
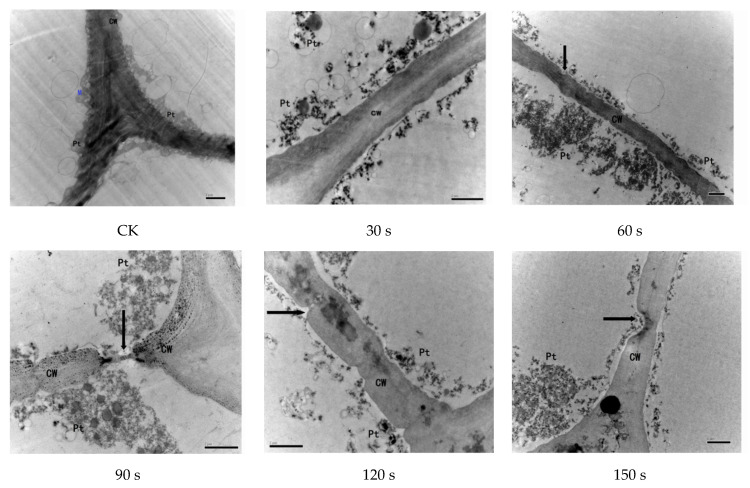
Transmission electron microscope (TEM) images of red pepper samples under different HHAIB treatment times. CK—control group; CW—cell wall; Pt—plastid; M—mitochondria.

Several recent studies have demonstrated that changes in microstructures can lead to changes in the macroscopic properties of organic matter [5,7,15]. Studying the changes in the microstructure of pigment peppers after HHAIB pretreatment is a powerful means of characterizing the drying speeds under different conditions [4]. The submicroscopic structures of pigment pepper skin cells at different processing times are shown in Figure 7. These TEM images show that untreated pigment pepper samples (CK) had smooth cell walls, a complete plasmid, and clear mitochondria and peroxisomes. With blanching, the peroxisomes and mitochondria rapidly decompose and eventually disappear [24]. The destruction of peroxisomes could effectively hinder the enzymatic browning reaction, which is good for the chemical protection of plants [28]. However, further blanching may lead to the collapse of the cell wall and cell tissue structures, which explains why the drying time increased with blanching duration [5].

## 4. Conclusions

The results show that pretreatment with HHAIB and drying with the specialized PVD equipment markedly affected the drying characteristics, color, and redness of peppers. Namely, HHAIB treatment shortened the drying time, reduced peroxidase activity, and had a significant impact on the water diffusion coefficient. During the drying process, an increase in the drying temperature shortened the drying time and improved color brightness. Blanching changed the cell structure of peppers and promoted the synthesis and release of chemical substances. The Page model presented the best fit for the drying kinetics of peppers. Thus, the study findings improve our understanding of the effects of HHAIB and PVD on the drying characteristics and changes in the color, red pigment content, and ultrastructure of peppers. Through HHAIB treatment, the drying time was shortened by 21.43% and the energy consumption of the drying process was reduced, whereas PVD improved the drying quality and effectively improved the quality and efficiency. It is recommended that industrial partners combine HHAIB with PVD in actual production to ensure minimum energy consumption and optimal quality. This study, thus, has good industrial application prospects and potential application value.

## Figures and Tables

**Table 1:** Moisture diffusion coefficient of pepper during the pulsed vacuum drying process at different blanching times estimated by the modified Weibull distribution function.

Blanching Time (s)	*D*_eff_ (10^−10^ m^2^/s)	*R* ^2^	Blanching Temperature (°C)	*D*_eff_ (10^−10^ m^2^/s)	*R* ^2^
Untreated	4.22 ^c^	0.9964	Untreated	4.22 ^c^	0.9964
30	4.62 ^b^	0.9978	110	4.71 ^b^	0.9973
60	4.71 ^b^	0.9973	120	4.82 ^b^	0.9918
90	5.01 ^a^	0.9956	130	5.11 ^a^	0.9886
120	4.66 ^b^	0.9927			
150	4.17 ^c^	0.9902			
180	3.89 ^d^	0.9895			

Notes: different letters in the figure reveal significant differences (*p* < 0.05) according to the Duncan test.

**Table 2:** Fitting parameters of different drying models.

	Two Term						
Condition	*k*_0_	*k*_1_	*a*	*b*	*R* ^2^	RMSE	*χ*^2^ × 10^4^
60 °C	3.031	−2.03	1.19	1.224	0.9983	0.01394	17.48
70 °C	3.672	−2.673	1.059	0.9177	0.9994	0.01246	0.000466
80 °C	10.22	−9.205	1.192	1.142	0.998	0.01986	15.77
	**Modified Weibull**						
Condition	*α*	*β*	*A*		*R* ^2^	RMSE	*χ*^2^ × 10^4^
60 °C	95.28	0.827	0.167		0.9864	0.03605	130
70 °C	84.72	0.998	0.192		0.9956	0.02391	28.6
80 °C	60	0.949	0.182		0.9947	0.03582	64.2
	**Wang and Singh**						
Condition	*a*	*b*			*R* ^2^	RMSE	*χ*^2^ × 10^4^
60 °C	−0.7027	0.122			0.9705	0.05189	296.2
70 °C	−0.8993	0.2067			0.9918	0.03364	67.91
80 °C	−1.21	0.365			0.9954	0.02472	36.66
	**Page**						
Condition	*k*	*n*			*R* ^2^	RMSE	*χ*^2^ × 10^4^
60 °C	1.116	1.03			0.9984	0.01221	16.41
70 °C	1.299	1.12			0.9978	0.01728	17.92
80 °C	1.893	1.276			0.9992	0.01002	6.026
	**Lewis**						
Condition	*k*				*R* ^2^	RMSE	*χ*^2^ × 10^4^
60 °C	1.126				0.9982	0.01227	18.06
70 °C	1.315				0.9957	0.02252	35.5
80 °C	1.794				0.9929	0.02846	56.71

**Table 3:** Effect of different drying temperature on color and red pigments.

Drying Temperature (°C)	Red Pigments (g/kg)	a*	b*	L*
60 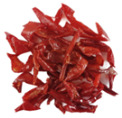	9.47 ± 0.12 ^b^	41.27 ± 0.05 ^b^	37.73 ± 0.15 ^c^	39.17 ± 0.07 ^c^
70 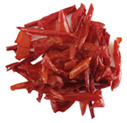	9.94 ± 0.18 ^a^	42.92 ± 0.06 ^a^	42.20 ± 0.07 ^b^	44.43 ± 0.18 ^a^
80 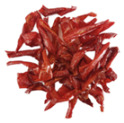	9.60 ± 0.06 ^b^	43.77 ± 0.02 ^a^	43.62 ± 0.02 ^a^	42.88 ± 0.09 ^b^

Notes: different letters in the figure reveal significant differences (*p* < 0.05) according to the Duncan test.

## Data Availability

The datasets generated for this study are available on request to the corresponding author.
